# Draft Genome Sequence of *Thermodesulfovibrio* sp. Strain Kuro-1, a Thermophilic, Lactate-Degrading Anaerobe Isolated from a Thermophilic Anaerobic Digestion Reactor

**DOI:** 10.1128/MRA.00709-19

**Published:** 2019-07-18

**Authors:** Takeshi Yamada, Jun Harada, Misaki Kurobe, Surya Giri, Masaru Konishi Nobu, Takashi Narihiro, Hideto Tsuji, Hiroyuki Daimon

**Affiliations:** a Department of Applied Chemistry and Life Science, Toyohashi University of Technology, Toyohashi, Japan; b Bioproduction Research Institute, National Institute of Advanced Industrial Science and Technology (AIST), Tsukuba, Japan; c Core for International Relations, Toyohashi University of Technology, Toyohashi, Japan; Broad Institute

## Abstract

*Thermodesulfovibrio* sp. strain Kuro-1, newly isolated from a thermophilic anaerobic digestion reactor, is a thermophilic anaerobe that can utilize l-lactic acid in fermentation, sulfate respiration, and cocultivation with hydrogenotrophic methanogens. Here, we report its draft genome sequence, consisting of a 1.93-Mb sequence with a G+C content of 34.0%.

## ANNOUNCEMENT

The genus *Thermodesulfovibrio* currently includes five species of thermophilic sulfate-reducing obligate anaerobic bacteria (1–3). Among these, T. yellowstonii, T. islandicus, and T. aggregans utilize l-lactic acid upon cocultivation with hydrogenotrophic methanogens (3). *Thermodesulfovibrio* spp. are common in thermophilic anaerobic digestion reactors for poly-l-lactic acid (PLLA) treatment using limited quantities of sulfate and sulfur compounds. However, the ecological niche of *Thermodesulfovibrio* spp. and other lactate-degrading bacteria in the reactor and the phenotypic and genotypic differences among *Thermodesulfovibrio* spp. remain unclear. Therefore, we isolated a novel *Thermodesulfovibrio* bacterium, strain Kuro-1, from a thermophilic PLLA treatment anaerobic digestion reactor using the roll-tube method involving serial dilutions (4). Strain Kuro-1 was found to be phylogenetically close to *T. yellowstonii* strain YP87^T^ and showed phenotypic similarities to strain YP87^T^ regarding metabolic processes, such as sulfate respiration and cocultivation with hydrogenotrophic methanogens. However, although strain Kuro-1 fermented l-lactic acid and pyruvate, strain YP87^T^ utilized only pyruvate (1). To further clarify these differences, we determined the draft genome sequence of strain Kuro-1.

Strain Kuro-1 was cultivated at 55°C for 14 days using basal medium (5) supplemented with 10 mM l-lactic acid. Bacterial DNA was extracted using proteinase K, as previously described (6), with the addition of phenol-chloroform treatment. A paired-end library was generated using the Kapa HyperPrep and FastGene adapter kits (Nippon Genetics, Tokyo, Japan) and sequenced using the NextSeq platform with 151-bp paired-end reads (Illumina, San Diego, CA, USA) at the Bioengineering Lab Co., Ltd. (Kanagawa, Japan). Of the 8,195,082 paired-end reads generated, low-quality reads were filtered out, and adaptors were removed using Trimmomatic v0.39 with the following parameters: sliding window, 6:30; minimum length, 78 (7). High-quality reads were assembled *de novo* using SPAdes v3.13.1 with k-mers of 19, 33, 47, 61, 75, 99, and 127 (8). The completeness of the draft genome was verified based on the abundance of single-copy marker genes specific for the phylum *Nitrospirae* using CheckM v1.0.7 (9). The draft genome was annotated using Prokka v1.12 (10) and functionally annotated using the Kyoto Encyclopedia of Genes and Genomes database (11).

The draft genome was found to consist of 11 contigs (>1,000 bp) with 640-fold average coverage. The sequence length is 1.93 Mb, and the G+C content is 34.0%. The completeness of the draft genome is 98.8%, and 668 genes were detected from the 676 single-copy marker genes. The draft genome sequence of strain Kuro-1 contains 1,937 protein-coding and 48 RNA-coding genes, including 1 encoding a transfer-messenger RNA (tmRNA) and 47 encoding tRNAs. Genes encoding key enzymes involved in sulfate respiration (sulfate adenylyltransferase, adenylyl-sulfate reductase, and dissimilatory sulfite reductase) were also identified. Further comparative genomic analyses of *Thermodesulfovibrio* spp. will provide insights into the ecophysiological traits of strain Kuro-1 in the thermophilic anaerobic digestion sludge used for PLLA treatment.

### Data availability.

This draft genome sequence was deposited at DDBJ/ENA/GenBank under accession number BJKS00000000. The version described in this paper is the first version, BJKS01000000. Raw sequencing reads are available in NCBI under the Sequence Read Archive accession number DRX173733.

## References

[1] Henry EA, Devereux R, Maki JS, Gilmour CC, Woese CR, Mandelco L, Schauder R, Remsen CC, Mitchell R 1994 Characterization of a new thermophilic sulfate-reducing bacterium *Thermodesulfovibrio yellowstonii*, gen. nov. and sp. nov.: its phylogenetic relationship to *Thermodesulfobacterium commune* and their origins deep within the bacterial domain. Arch Microbiol 161:62–69. doi:10.1007/BF00248894. 11541228

[2] Sonne-Hansen J, Ahring BK 1999 *Thermodesulfobacterium hveragerdense* sp. nov. and *Thermodesulfovibrio islandicus* sp. nov., two thermophilic sulfate reducing bacteria isolated from a Icelandic hot spring. Syst Appl Microbiol 22:559–564. doi:10.1016/S0723-2020(99)80009-5. 10794144

[3] Sekiguchi Y, Muramatsu M, Imachi H, Narihiro T, Ohashi A, Harada H, Hanada S, Kamagata Y 2008 *Thermodesulfovibrio aggregans* sp. nov. and *Thermodesulfovibrio thiophilus* sp. nov., anaerobic, thermophilic, sulfate-reducing bacteria isolated from thermophilic methanogenic sludge, and emended description of the genus *Thermodesulfovibrio*. Int J Syst Evol Microbiol 58:2541–2548. doi:10.1099/ijs.0.2008/000893-0. 18984690

[4] Hungate RE 1969 Chapter IV a roll tube method for cultivation of strict anaerobes. Methods Microbiol 3:117–132. doi:10.1016/S0580-9517(08)70503-8.

[5] Widdel F, Pfenning N 1981 Studies on dissimilatory sulfate-reducing bacteria that decompose fatty acids. I. Isolation of new sulfate-reducing bacteria enriched with acetate from saline environments. Description of *Desulfobacter postgatei* gen. nov., sp. nov. Arch Microbiol 129:395–400. doi:10.1007/BF00406470. 7283636

[6] Hiraishi A 1992 Direct automated sequencing of 16S rDNA amplified by polymerase chain reaction from bacterial cultures without DNA purification. Lett Appl Microbiol 15:210–213. doi:10.1111/j.1472-765X.1992.tb00765.x. 1280147

[7] Bolger AM, Lohse M, Usadel B 2014 Trimmomatic: a flexible trimmer for Illumina sequence data. Bioinformatics 30:2114–2120. doi:10.1093/bioinformatics/btu170. 24695404 PMC4103590

[8] Bankevich A, Nurk S, Antipov D, Gurevich AA, Dvorkin M, Kulikov AS, Lesin VM, Nikolenko SI, Pham S, Prjibelski AD, Pyshkin AV, Sirotkin AV, Vyahhi N, Tesler G, Alekseyev MA, Pevzner PA 2012 SPAdes: a new genome assembly algorithm and its applications to single-cell sequencing. J Comput Biol 19:455–477. doi:10.1089/cmb.2012.0021. 22506599 PMC3342519

[9] Parks DH, Imelfort M, Skennerton CT, Hugenholtz P, Tyson GW 2015 Assessing the quality of microbial genomes recovered from isolates, single cells and metagenomes. Genome Res 25:1043–1055. doi:10.1101/gr.186072.114. 25977477 PMC4484387

[10] Seemann T 2014 Prokka: rapid prokaryotic genome annotation. Bioinformatics 30:2068–2069. doi:10.1093/bioinformatics/btu153. 24642063

[11] Kanehisa M, Goto S 2000 KEGG: Kyoto Encyclopedia of Genes and Genomes. Nucleic Acids Res 28:27–30. doi:10.1093/nar/28.1.27. 10592173 PMC102409

